# Effect of Nurse-Based Management of Hypertension in Rural Western Kenya

**DOI:** 10.5334/gh.856

**Published:** 2020-12-01

**Authors:** Rajesh Vedanthan, Anirudh Kumar, Jemima H. Kamano, Helena Chang, Samantha Raymond, Kenneth Too, Deborah Tulienge, Charity Wambui, Emilia Bagiella, Valentin Fuster, Sylvester Kimaiyo

**Affiliations:** 1 Department of Population Health, NYU Grossman School of Medicine, New York, US; 2 Department of Medicine, NYU Grossman School of Medicine, New York, US; 3 Department of Medicine, School of Medicine, Moi University College of Health Sciences, Eldoret, KE; 4 Chronic Disease Management, Academic Model Providing Access to Healthcare, Eldoret, KE; 5 Department of Population Health Science and Policy, Icahn School of Medicine at Mount Sinai, New York, US; 6 Mount Sinai Heart, Icahn School of Medicine at Mount Sinai, New York, US

**Keywords:** blood pressure, hypertension, nurse management, task redistribution, global health, low- and middle-income countries

## Abstract

**Background:**

Elevated blood pressure is the leading cause of death worldwide; however, treatment and control rates remain very low. An expanding literature supports the strategy of task redistribution of hypertension care to nurses.

**Objective:**

We aimed to evaluate the effect of a nurse-based hypertension management program in Kenya.

**Methods:**

We conducted a retrospective data analysis of patients with hypertension who initiated nurse-based hypertension management care between January 1, 2011, and October 31, 2013. The primary outcome measure was change in systolic blood pressure (SBP) over one year, analyzed using piecewise linear mixed-effect models with a cut point at 3 months. The primary comparison of interest was care provided by nurses versus clinical officers. Secondary outcomes were change in diastolic blood pressure (DBP) over one year, and blood pressure control analyzed using a zero-inflated Poisson model.

**Results:**

The cohort consisted of 1051 adult patients (mean age 61 years; 65% women). SBP decreased significantly from baseline to three months (nurse-managed patients: slope –4.95 mmHg/month; clinical officer-managed patients: slope –5.28), with no significant difference between groups. DBP also significantly decreased from baseline to three months with no difference between provider groups. Retention in care at 12 months was 42%.

**Conclusions:**

Nurse-managed hypertension care can significantly improve blood pressure. However, retention in care remains a challenge. If these results are reproduced in prospective trial settings with improvements in retention in care, this could be an effective strategy for hypertension care worldwide.

## Introduction

Elevated blood pressure (BP), a major risk factor for ischemic heart disease [1], heart failure [2], and stroke [3], is the leading global risk for mortality [4]. While BP control reduces cardiovascular disease and death [5,6], treatment and control rates are suboptimal worldwide [7]. The economic burden of hypertension is staggering, in both direct health costs and indirect impact on productivity [8,9]. Unless adequately controlled, hypertension will continue to be responsible for significant health and economic burden worldwide.

Recent literature suggests that health care worker density is significantly associated with hypertension treatment rates, and that nurse density in particular appears to be an important determinant [10]. Given the severe shortage of physicians in LMICs, task redistribution of hypertension care from physicians to nurses could improve hypertension treatment and control rates in low-resource settings [11]. Task redistribution is a strategy in which specific tasks are ‘redistributed’ among health workers with varying levels and duration of training [12]. There is a growing literature examining the effectiveness of task redistribution from physicians to non-physicians for the treatment of hypertension [13]. While promising, nurse management of hypertension in LMICs remains evaluated in a limited number of settings to date.

In 2011, Academic Model Providing Access to Healthcare (AMPATH) Chronic Disease Management Program initiated nurse-based hypertension management in rural western Kenya [14]. The aim of this study is to determine the impact of this program on BP change, by performing secondary analysis of routinely collected clinical data. We anticipate that this will help inform the evaluation of task redistribution as an essential strategy to meet the human resource challenge of chronic disease management in Kenya and other LMICs.

## Methods

### Setting

**Figure 1:**
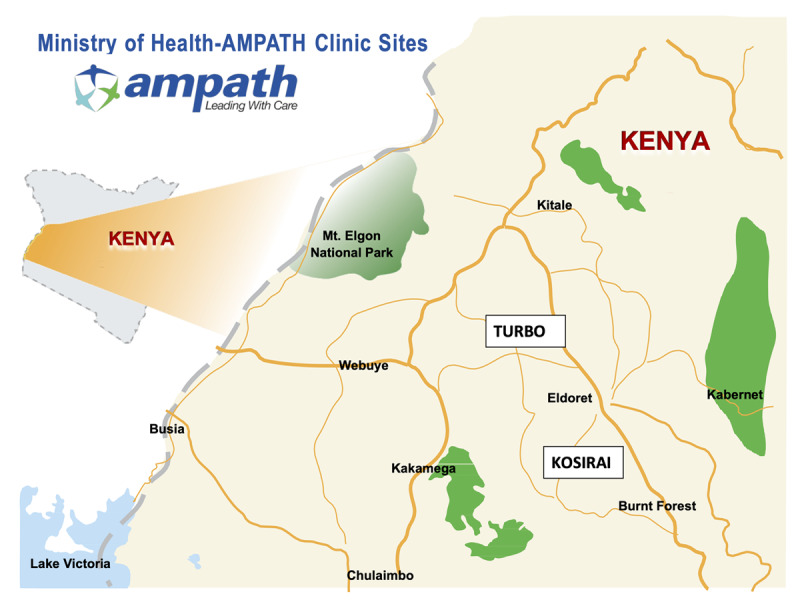
Catchment area of the study in western Kenya, with Turbo and Kosirai Divisions highlighted.

The AMPATH program is an academic global health partnership between Moi Teaching and Referral Hospital, Moi University College of Health Sciences, and a consortium of North American universities led by Indiana University [15]. AMPATH was established in Kenya in 2001, and developed a geographically decentralized, community-based HIV care system in Western Kenya that has served over 200,000 patients [16]. Building upon this foundation, and in recognition of the growing NCD burden, AMPATH established a Chronic Disease Management Program in collaboration with the Ministry of Health and expanded its clinical scope of work to address comprehensive primary care, including hypertension [14]. The Chronic Disease Management Program provides clinical care in over 150 health facilities spanning all levels of the public sector health care system in western Kenya. This study was conducted within the AMPATH catchment area, specifically in the Kosirai and Turbo Divisions (Figure 1). In these geographical areas for the time period of this study, there were twenty rural dispensaries staffed primarily by nurses, as well as two rural health centers staffed primarily by clinical officers (mid-level practitioners). We embedded this study within an ongoing project aimed at evaluating the feasibility and impact of nurse management of hypertension in Africa [17].

### Design

This study is a retrospective analysis of routine clinical data collected by the AMPATH Chronic Disease Management Program. All individuals enrolled in this nurse-based hypertension management program received the same care, according to standardized AMPATH protocols based on the contemporaneous evidence-based standards of JNC-7 and JNC-8 (Appendix A) [18,19], which include lifestyle counseling, nurse prescription of initial medication, clear algorithmic criteria for escalation of pharmacotherapy and frequency of return clinical visits, and referral to higher care as required. All clinicians received initial training regarding the management of hypertension, ongoing mentorship on a regular basis, and clinical decision support (either paper-based or smartphone-based [20]). Medications were reliably and continuously supplied by either the Kenya Ministry of Health facility pharmacy or backed up by the AMPATH revolving fund pharmacy program [21,22]. Patients procured their medicines within the premises of the Ministry of Health facility where the clinical encounter occurred. Standard user fees for clinical care and medications were negotiated between the AMPATH Chronic Disease Management Program and the Kenyan Ministry of Health (Appendix A). Active linkage and retention activities were part of the program, involving community health workers reminding patients to return to clinic if they missed an appointment [23].

### Participants

Community-based screening of all adults ≥18 years for systolic blood pressure (SBP) and diastolic blood pressure (DBP), using automatic BP machines, was initiated by AMPATH in both Kosirai and Turbo Divisions [24]. The screening activities included both home-based screening and community-wide screening events, and screening was conducted by trained community health workers and Chronic Disease Management Program staff. Individuals with an initial elevated BP (SBP ≥ 140 or DBP ≥ 90) were referred to the local dispensary for further evaluation and confirmation. At the dispensary, each individual had his/her BP measured again by the clinician (either nurse or clinical officer), in order to confirm the diagnosis of hypertension (defined as SBP ≥ 140 or DBP ≥ 90). Individuals with confirmed hypertension were enrolled into the AMPATH Chronic Disease Management Program. Individuals who had symptoms of complications or high-risk features were referred directly to higher levels of care at the Health Center or Referral Hospital, to be seen by either clinical officers or physicians, respectively. Individuals enrolled in nurse-based management received their care at the nearest rural dispensary or at the rural health center contingent on geographical proximity, and are the subject of this current report. Individuals being seen at the health center were often seen by a clinical officer due to staffing considerations. Clinical mentorship of the nurses was provided by clinical officers and physicians in the AMPATH Chronic Disease Management Program.

### Procedures

Routine, de-identified, clinical data within the AMPATH Medical Record System (AMRS) were extracted without personal identifiers. Hence, informed consent from patients was not required. The clinical cohort was comprised of hypertensive patients over the age of 35 who initiated care between January 1, 2011, and October 31, 2013. The follow-up period was one year (+/– 3 months), and data were extracted from the electronic medical record system through December 31, 2014. Data extracted included: sex, age, encounter type, encounter date, SBP, DBP, facility type, provider type (clinical officer or nurse), and health center location (Kosirai or Turbo Division). BP measurements were conducted and recorded into the medical record by the clinical provider. Errant BP values per AMPATH Chronic Disease Management data management guidelines (SBP < 50 or SBP > 250; DBP < 30 or DBP > 150) were excluded from the analysis. In addition, clinical encounters with either a missing SBP or DBP value were also excluded, since it was unlikely that only one of the BP parameters was measured during the clinical encounter.

### Statistical Analysis

The primary outcome measure was changed in SBP over one year, analyzed using piecewise linear mixed-effect models with random intercept and slopes. The response curve was divided into two segments with a linear model having different slopes fitted to each segment. The location where the two segments joined, called a ‘knot,’ was placed at three months to reflect clinical reality in terms of frequency of a return visit. The primary non-randomized intervention effect of interest was a comparison of SBP change among patients cared for by nurses versus those cared for by clinical officers. Within- and between-group changes before and after three months from baseline were assessed. Additional covariates included age, sex, and health center location. Age was mean-centered.

The key secondary outcomes included change in DBP over one year and the frequency of BP control over one year. DBP was analyzed using the same method as described for change in SBP. BP control was defined as having both a SBP < 140 and a DBP < 90 during a clinical encounter. Only patients with more than one visit (and therefore opportunity for BP control) were included in all BP control analyses. BP control was analyzed using a zero-inflated Poisson model to account for individuals who never had their blood pressure controlled. The two-part modeling of the zero-inflated Poisson regression provided the effect of provider on lack of any BP control (a ‘zero’) and the effect of provider on the relative frequency of BP control over time among those who were ever controlled. Logistic regression was used to model the probability of lack of BP control and Poisson regression was used to model mean number of times BP was controlled. The Poisson model included a covariate for the (log) number of visits to account for variation in number of visits. Covariates included mean-centered age, sex, and health center location.

As a sensitivity analysis, SBP and DBP analyses were run only on patients with more than one visit. All analyses were performed at the 0.05 (2-sided) significance level using SAS Version 9.4 (SAS Institute, North Carolina, USA).

## Results

Of the 1432 patients enrolled in the Chronic Disease Management Program during the study period, 1051 individuals (65% women) were over age 35 and were confirmed as hypertensive by the clinician. Of these 1051 individuals, 753 (72%) had at least one follow-up clinical visit during the 12-month follow-up period and 446 patients (42%) were retained in care at one year (+/– 3 months). Women were more likely than men to remain in care at 12 months (45% vs. 38%).

Of the entire clinical cohort, 180 individuals were cared for by a nurse and 871 were cared for by a clinical officer. The mean age was 60.6 years (SD 13.2), with no significant difference between those managed by a nurse versus a clinical officer (Table 1). Patients seen in the health center were predominantly managed by a clinical officer, while those seen in the dispensary were managed exclusively by a nurse. Few patients switched level of provider over the follow-up period (14 patients were initially seen by a nurse and then subsequently by a clinical officer, while 34 patients were initially seen by a clinical officer and then subsequently by a nurse). There was no clearly detectable BP pattern associated with either referral up or referral down. Baseline average SBP and DBP were 166.2 mmHg (SD 22.2) and 95.8 mmHg (SD 13.3), respectively, with no significant baseline difference between those managed by a nurse versus clinical officer.

**Table 1:** Characteristics of the study population, divided into those primarily managed by a nurse versus clinical officer.

	Nurse (N = 180)	Clinical Officer (N = 871)	Total (N = 1051)	P Value

Age in years, mean ± SD	60.1 ± 13.9	60.7 ± 13.0	60.6 ± 13.2	0.58
Gender				0.84
Female (%)	119 (66.1)	569 (65.3)	688 (65.5)	
Male (%)	61 (33.9)	302 (34.7)	363 (34.5)	
Facility type				<0.0001
Health center (%)	4 (2.2)	871 (100)	875 (83.3)	
Dispensary (%)	176 (97.8)	0 (0)	176 (16.7)	
Health center				<0.0001
Kosirai (%)	36 (20.0)	571 (65.6)	607 (57.8)	
Turbo (%)	144 (80.0)	300 (34.4)	444 (42.2)	
Baseline SBP in mmHg, mean ± SD	166.2 ± 22.2	166.1 ± 27.9	166.1 ± 27.0	0.94
Baseline DBP in mmHg, mean ± SD	95.8 ± 13.3	96.3 ± 14.5	96.2 ± 14.3	0.66

**Figure 2:**
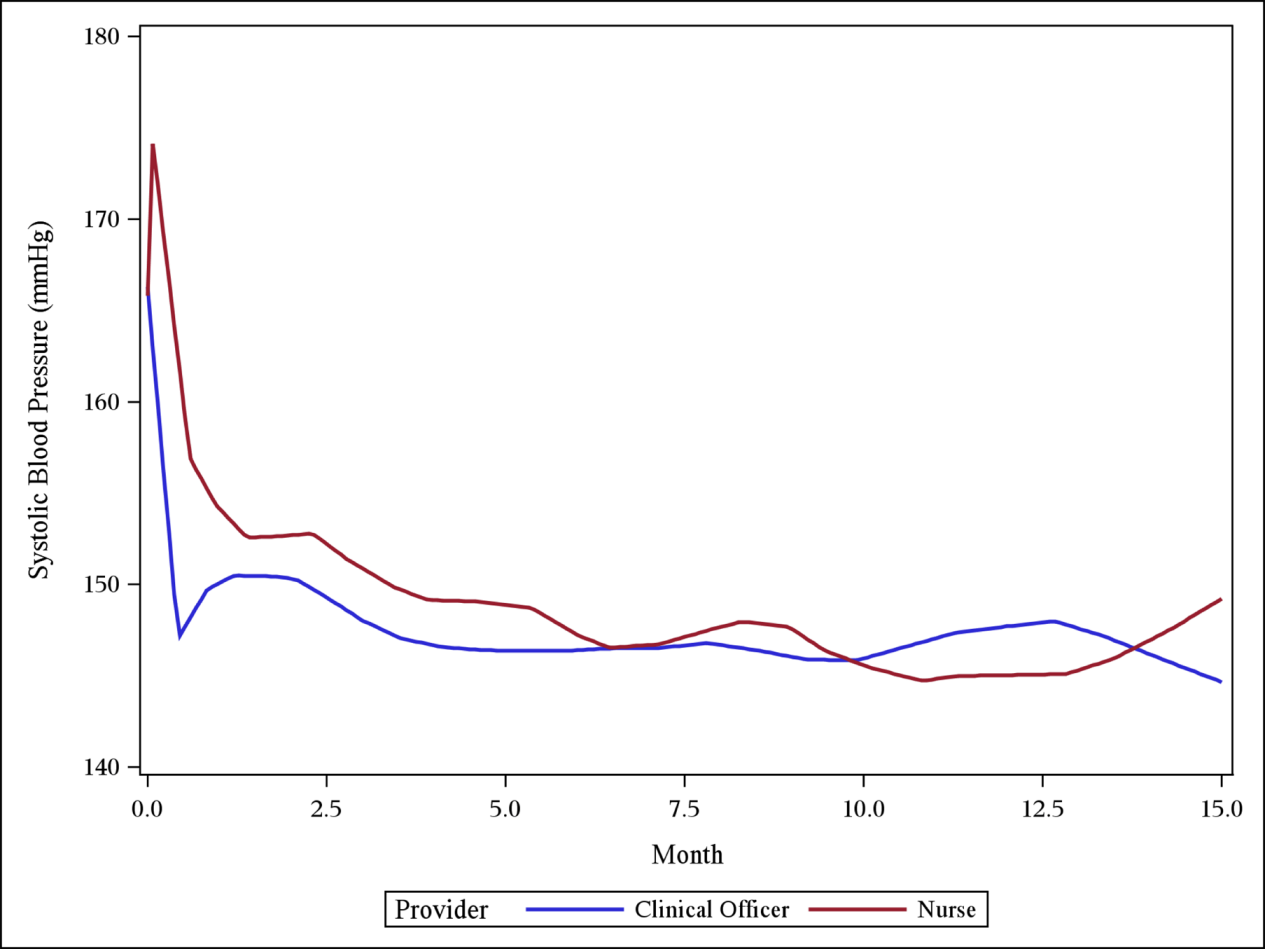
Loess plot of mean SBP over time, demonstrating early reduction in SBP in both the nurse and clinical officer groups, maintained during the follow-up period.

SBP declines were achieved early within the first few months for both nurse-managed and clinical officer-managed patients, and maintained throughout the follow-up period (Figure 2). After adjustment for age, sex, and health center location, both nurse-managed and clinical officer-managed patients experienced significant reduction in SBP from baseline to three months (nurse-managed: slope –4.95 mmHg/month; 95% CI –6.55 to –3.35; clinical officer-managed: slope –5.28; –5.99 to –4.57) (Table 2). This is equivalent to 15 mmHg reduction in SBP over three months, with no significant different between groups (difference in slopes 0.33; –1.42 to 2.08). SBP remained relatively unchanged after three months (nurse-managed: slope –0.44; –0.97 to 0.10; clinical officer-managed patients: slope 0.17; –0.07 to 0.40). While nurse-managed patients had a more favorable slope than the clinical officer- managed patients after three months, there was sparsity and spread of data over time due to the poor retention in care. These slope values translate into an approximately 18.8 mmHg SBP reduction over the entire 12 months for nurse-managed patients. There was no difference between men and women with respect to SBP lowering. Older patients, relative to younger patients, tended to have less SBP reduction over the follow-up period. Patients in the Turbo Division experienced greater SBP reduction than patients in Kosirai.

**Table 2:** Results of the piecewise linear spline model for SBP change over time, with knot at three months. Model parameter estimates based on piecewise linear mixed-effect models with random intercept and slopes and a knot placed at three months, adjusted by age, sex, and healthcare center. The estimate values indicate the absolute change in SBP (mmHg) per month, except for the ‘other variables,’ which are for the entire follow-up period.

	Estimate (95% CI)	P Value

**Slope before three months**		
Nurse	–4.95 (–6.55 to –3.35)	<0.0001
Clinical Officer	–5.28 (–5.99 to –4.57)	<0.0001
Nurse-Clinical Officer	0.33 (–1.42 to 2.08)	0.71
**Slope after three months**		
Nurse	–0.44 (–0.97 to 0.10)	0.11
Clinical Officer	0.17 (–0.07 to 0.40)	0.16
Nurse-Clinical Officer	–0.60 (–1.19 to –0.02)	0.04
**Change in slope from before to after three months**		
Nurse	4.51 (2.54 to 6.49)	<0.0001
Clinical Officer	5.45 (4.58 to 6.31)	<0.0001
Nurse-Clinical Officer	–0.93 (–3.09 to 1.22)	0.40
**Other variables**		
Age (per year)	0.23 (0.14 to 0.32)	<0.0001
Sex (men vs. women)	0.84 (–1.67 to 3.35)	0.5122
Facility (Turbo vs. Kosirai)	–6.83 (–9.29 to –4.38)	<0.0001

DBP, in a similar manner to SBP, declined within the first few months and then was maintained throughout follow-up (Supplemental Figure). After adjustment for age, sex, and health center location, both nurse-managed and clinical officer-managed patients experienced significant reduction in DBP from baseline to three months (nurse-managed: slope –2.37; 95% CI –3.24 to –1.50; clinical officer-managed: slope –2.45; 95% CI –2.38 to –2.07), and the decrease was not significantly different between provider groups (difference in slopes 0.08; 95% CI –0.87 to 1.02) (Supplemental Table 1). DBP remained relatively unchanged after three months (nurse-managed: slope –0.28; 95% CI –0.58 to 0.01; clinical officer-managed: slope 0.07; 95% CI –0.06 to 0.20). There was no difference between men and women with respect to DBP lowering. Older patients, relative to younger patients, tended to have greater DBP reduction over the follow-up period. Patients in the Turbo Division experienced greater DBP reduction than patients in Kosirai. SBP and DBP sensitivity analyses were also performed only on patients with more than one visit (n = 753), which produced very similar results (Supplemental Tables 2 and 3).

BP control analyses were also conducted on the subset of patients with more than one visit. After adjusting for age, sex, and health center location, there was no difference in the proportion of patients who never had their blood pressure controlled between nurses and clinical officers (50.0% vs 38.0%, respectively; odds ratio 1.32, 95% CI 0.57 to 3.09) (Table 3). Among patients who ever achieved BP control, the frequency of maintaining control was lower in patients managed by nurses compared to patients managed by clinical officers (rate ratio 0.80, 95% CI, 0.67 to 0.97). There was no difference between men and women, or by age group, with respect to BP control.

**Table 3:** Results of the zero-inflated Poisson model for BP control over time. Model parameter estimates based on a zero-inflated Poisson model adjusted by age, sex, and healthcare center. Only patients with more than one visit are included (n = 753).

Zero-Inflated Model	OR (95% CI)	P Value

Nurse vs. Clinical Officer	1.32 (0.57, 3.09)	0.52
**Poisson Model**	**RR (95% CI)**	**P Value**

Nurse vs. Clinical Officer	0.80 (0.67, 0.97)	0.02

## Discussion

In this study, we were able to demonstrate that AMPATH’s nurse-based hypertension management program resulted in significant SBP and DBP reduction over a 12-month follow-up period. The SBP and DBP declines were largely observed within the first three months of clinical follow-up, and these changes were maintained over time. No statistical differences were observed in the SBP and DBP reductions from baseline to the three-month mark, between nurse- and clinical officer-managed patients. Nurse-managed patients experienced slightly greater SBP reduction after the three-month mark, although there was sparsity and spread of data particularly as the follow-up period increased.

These results are consistent with the findings of other similar studies in other parts of the world. A program of nurse-led hypertension management based in urban and rural Cameroon showed an average decline in SBP of 11.7 mmHg over 26 months [25]. A recent trial from Ghana demonstrated that nurse-led management of hypertension was superior to usual care [26]. Another study of primary health facilities in Kibera, Kenya, demonstrated that nurses were able to adhere to clinical protocols for NCD management, thereby reaffirming the feasibility of task redistribution for hypertension care in a very similar milieu [27].

While there were no differences between nurse and clinical officer management with respect to the proportion of patients who ever achieved BP control, the maintenance of BP control was lower in patients managed by nurses. This finding is not inconsistent with the clinical protocols implemented by the AMPATH Chronic Disease Program, which recommended referral to a clinical officer if BP remained challenging to control only after three consecutive monthly visits with elevated BP. Despite significant reductions in SBP and DBP, achieving BP control proved challenging. This challenge has been confronted by similar projects in other LMICs. A World Health Organization study based in primary care facilities in China and Nigeria achieved control rates of 45.2% and 38.1%, respectively [28]. A study in the slums of Nairobi reported that only 21.5% of individuals on treatment had controlled BP control [29]. The LARK study from western Kenya also yielded low control rates despite substantial BP reductions [23]. Nevertheless, given the substantive and significant absolute declines in SBP observed in our study, substantial cardiovascular and mortality benefit would be expected from both clinical and population perspectives [6].

Loss to follow-up was also a notable challenge in our study. Only 42% of the patients were retained in care at the 12-month mark, and women were more likely to remain in care than men. This highlights that although nurse management of hypertension can improve BP, other strategies must be implemented to increase retention to care within this setting. Potential causes for diminished follow-up could have been socioeconomic stressors (unemployment, poverty, food insecurity, volatile family life), poor provider-patient relationship, the need for technical and mobility assistance to attend appointments, and general distrust of the medical enterprise [30]. While the use of community health workers and mobile health technology in western Kenya did improve linkage to hypertension care and a trend toward greater SBP reduction, the SBP difference was not statistically significant compared to usual care [23]. Addressing financial barriers [31], improving provider-patient communication [32], and enhancing patient education [33] are important areas of future study and intervention. Ensuring standard quality of care across all health facilities and geographic areas will be critical for future program implementation. In addition, incorporating sex- and gender-specific differences, preferences, and patterns will be critical to ensure the success of future programs with respect to the issue of retention in care.

A central principle of this program was leveraging the existing infrastructure and task redistribution strategy implemented by AMPATH for the treatment and prevention of HIV [14]. In addition to task redistribution, the AMPATH Chronic Disease Management Program involves a multicomponent care package that includes clinical decision support using health information technology [34], consistent and secure medication supply [22], linkage and retention activities [23], community and stakeholder engagement [35], and social support for patients. This multicomponent package aligns with all of the World Health Organization health system building blocks: service delivery, health workforce, health information systems, access to essential medicines, financing, and leadership/governance [36]. Indeed, addressing potential health system barriers and leveraging health system facilitators will help to optimize the success of task redistribution and related strategies [37].

One of the primary limitations of this study was the lack of a controlled comparator group. The primary reason for this limitation is that, in the absence of this hypertension program, rural patients would otherwise have no access to hypertension care other than at the District and Referral Hospitals. Thus, the AMPATH Chronic Disease Management Program leadership felt that it would be unethical to monitor outcomes in a controlled comparator group without offering those individuals a care program that was geographically accessible and decentralized. In addition, the results of this impact evaluation can serve as the foundation to design and conduct a larger scale trial to rigorously test the hypothesis that BP control in hypertensive patients can be achieved by nurses as effectively as by clinical officers and physicians in this setting. Another limitation is related to the implementation of the hypertension management program; nurses were routinely mentored by clinical officers and physicians, in order to ensure quality of care. However, those mentorship sessions often evolved into the care primarily being driven by the mentors rather than the nurses. We therefore separated the cohort into individuals whose care was primarily by a nurse versus clinical officer, and were able to demonstrate that nurse-managed patients fared just as well and potentially even modestly better in terms of SBP and DBP. Finally, this analysis was limited to the initial implementation period of the program, and our team has been able to increase geographic decentralization over time in alignment with task redistribution. Future analyses will be necessary that take into account program scale-up, program maturation, and lessons learned.

## Conclusion

Hypertension is a growing risk worldwide, particularly in LMICs. This study has demonstrated that nurses working within a resource-limited setting can successfully manage hypertension and achieve significant reductions in BP. Although combating hypertension and other non-communicable diseases will require a multifaceted approach, the nurse management of hypertension model described here can serve as a model for similar non-communicable disease care delivery programs in other low-resource settings worldwide. If the findings of this study are reproduced in prospective trial settings with improvements in retention in care, nurse management and task redistribution could be an effective strategy for improving hypertension outcomes and addressing this growing global public health concern.

## Data Accessibility Statement

The datasets used and/or analyzed during the current study are available on reasonable request to the corresponding author and the AMPATH Research Manager. This study complies with the NIH Public Access Policy, which ensures that the public has access to the published results of NIH-funded research, and therefore, all results have been (and will be made) available from final peer-reviewed journal manuscripts (including this one) via the digital archive PubMed Central upon acceptance for publication.

## Additional Files

The additional files for this article can be found as follows:

10.5334/gh.856.s1 Supplemental Figure. Loess plot of mean DBP over time, demonstrating early reduction in DBP in both the nurse and clinical officer groups, maintained during the follow-up period.

10.5334/gh.856.s2 Supplemental Table 1. Results of the piecewise linear spline model for DBP change over time, with knot at three months. The estimate values indicate the absolute change in DBP (mmHg) per month, except for the ‘other variables,’ which are for the entire follow-up period.

10.5334/gh.856.s3 Supplemental Table 2. Piecewise linear spline model for SBP change over time, using data for patients with more than one clinical visit.

10.5334/gh.856.s4 Supplemental Table 3. Piecewise linear spline model for DBP change over time, using data for patients with more than one clinical visit.

10.5334/gh.856.s5 Appendix A. AMPATH Chronic Disease Management hypertension management protocol.
