# Supplementary material for: Effect of Nurse-Based Management of Hypertension in Rural Western Kenya
Source: Glob Heart. 2020 Dec 1;15(1):77. doi: 10.5334/gh.856 (PMC7716784; doi:10.5334/gh.856)

S1. Supplemental Figure. Loess plot of mean DBP over time, demonstrating early reduction in DBP in both the nurse and clinical officer groups, maintained during the follow-up period.

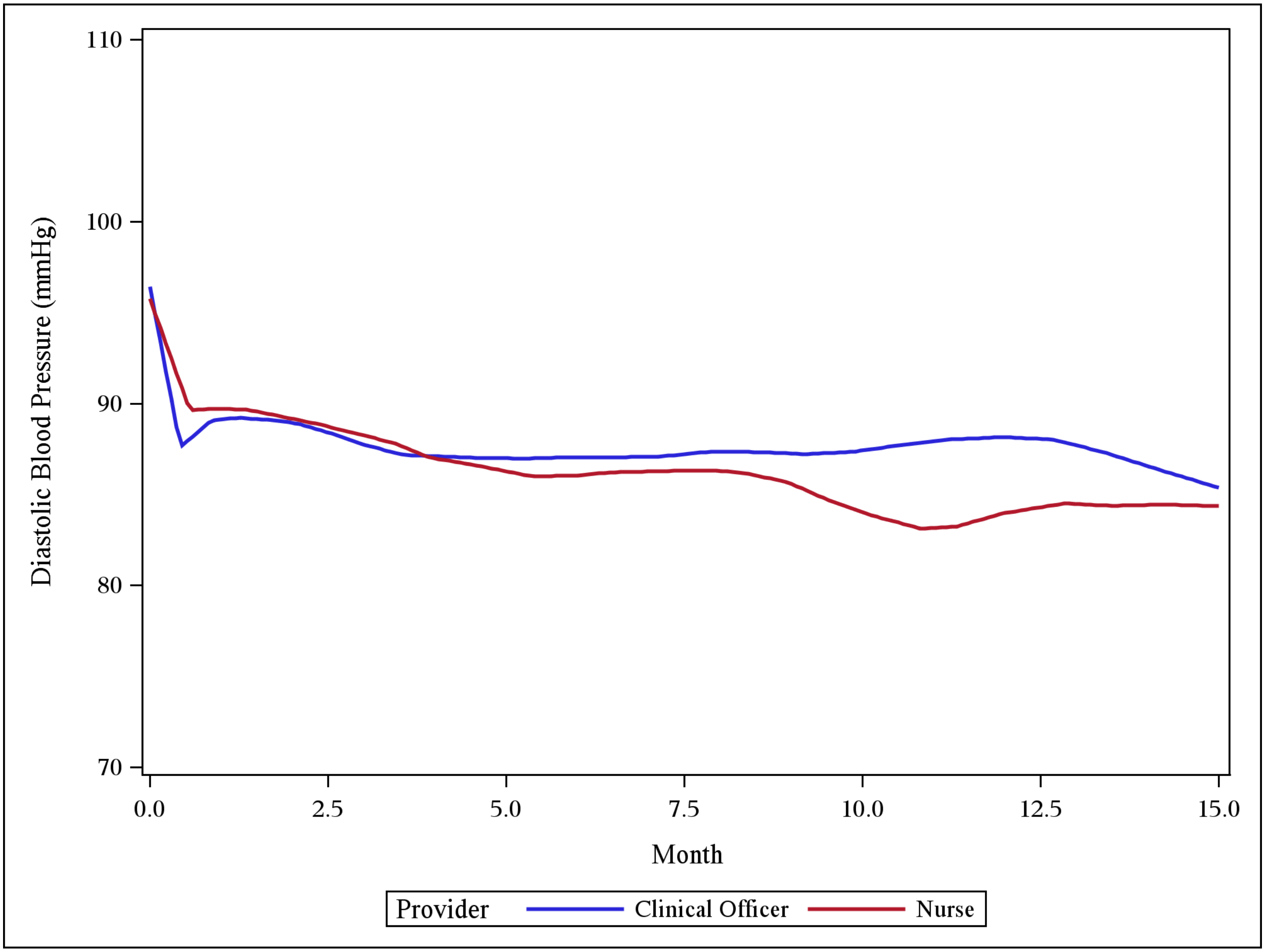

Supplement: Supplemental Figure. — Loess plot of mean DBP over time, demonstrating early reduction in DBP in both the nurse and clinical officer groups, maintained during the follow-up period. [file gh-15-1-856-s1.pdf]
