# Supplementary material for: Effect of Nurse-Based Management of Hypertension in Rural Western Kenya
Source: Glob Heart. 2020 Dec 1;15(1):77. doi: 10.5334/gh.856 (PMC7716784; doi:10.5334/gh.856)
Supplement: Supplemental Table 1. — Results of the piecewise linear spline model for DBP change over time, with knot at three months. The estimate values indicate the absolute change in DBP (mmHg) per month, except for the ‘other variables,’ which are for the entire follow-up period. [file gh-15-1-856-s2.pdf]

S2. Supplemental Table 1. Slope of change in diastolic blood pressure, before and after three months after initiating care.

	Estimate (95% CI)	P Value
**Slope before 3 months**		
Nurse	-2.37 (-3.24 to -1.50)	<.0001
Clinical Officer	-2.45 (-2.83 to -2.07)	<.0001
Nurse - Clinical Officer	0.08 (-0.87 to 1.02)	0.8748
**Slope after 3 months**		
Nurse	-0.28 (-0.58 to 0.01)	0.0596
Clinical Officer	0.07 (-0.06 to 0.20)	0.2774
Nurse - Clinical Officer	-0.35 (-0.67 to -0.03)	0.0307
**Change in slope from before to after 3 months**		
Nurse	2.09 (1.01 to 3.16)	<.0001
Clinical Officer	2.52 (2.06 to 2.98)	<.0001
Nurse - Clinical Officer	-0.43 (-1.60 to 0.74)	0.4714

Model parameter estimates based on piecewise linear mixed-effect models with random intercept and slopes and a knot placed at 3 months, adjusted by age, sex, and healthcare center.
