# Supplementary material for: Effect of Nurse-Based Management of Hypertension in Rural Western Kenya
Source: Glob Heart. 2020 Dec 1;15(1):77. doi: 10.5334/gh.856 (PMC7716784; doi:10.5334/gh.856)
Supplement: Supplemental Table 2. — Piecewise linear spline model for SBP change over time, using data for patients with more than one clinical visit. [file gh-15-1-856-s3.pdf]

S3. Supplemental Table 2. Systolic Blood Pressure among Patients with Multiple Visits

	Estimate (95% CI)	P Value
**Slope before 3 months**		
Nurse	-4.84 (-6.50 to -3.18)	<.0001
Clinical Officer	-5.08 (-5.82 to -4.33)	<.0001
Nurse - Clinical Officer	0.24 (-1.58 to 2.05)	0.7976
**Slope after 3 months**		
Nurse	-0.44 (-0.98 to 0.09)	0.1059
Clinical Officer	0.16 (-0.07 to 0.39)	0.1802
Nurse - Clinical Officer	-0.60 (-1.18 to -0.02)	0.0433
**Change in slope from before to after 3 months**		
Nurse	4.40 (2.37 to 6.43)	<.0001
Clinical Officer	5.24 (4.34 to 6.13)	<.0001
Nurse - Clinical Officer	-0.84 (-3.05 to 1.38)	0.4578

Model parameter estimates based on piecewise linear mixed-effect models with random intercept and slopes and a knot placed at 3 months
