# Supplementary material for: Effect of Nurse-Based Management of Hypertension in Rural Western Kenya
Source: Glob Heart. 2020 Dec 1;15(1):77. doi: 10.5334/gh.856 (PMC7716784; doi:10.5334/gh.856)
Supplement: Supplemental Table 3. — Piecewise linear spline model for DBP change over time, using data for patients with more than one clinical visit. [file gh-15-1-856-s4.pdf]

S4. Supplemental Table 3. Diastolic Blood Pressure among Patients with Multiple Visits

	Estimate (95% CI)	P Value
**Slope before 3 months**		
Nurse	-2.33 (-3.23 to -1.44)	<.0001
Clinical Officer	-2.31 (-2.71 to -1.92)	<.0001
Nurse - Clinical Officer	-0.02 (-1.00 to 0.96)	0.9690
**Slope after 3 months**		
Nurse	-0.28 (-0.58 to 0.01)	0.0593
Clinical Officer	0.07 (-0.06 to 0.19)	0.3017
Nurse - Clinical Officer	-0.35 (-0.67 to -0.03)	0.0323
**Change in slope from before to after 3 months**		
Nurse	2.05 (0.95 to 3.15)	<.0001
Clinical Officer	2.38 (1.90 to 2.86)	<.0001
Nurse - Clinical Officer	-0.33 (-1.53 to 0.87)	0.5886

Model parameter estimates based on piecewise linear mixed-effect models with random intercept and slopes and a knot placed at 3 months. Three DBP observations > 150 were removed.
