# Supplementary material for: Effect of Nurse-Based Management of Hypertension in Rural Western Kenya
Source: Glob Heart. 2020 Dec 1;15(1):77. doi: 10.5334/gh.856 (PMC7716784; doi:10.5334/gh.856)
Supplement: Appendix A. — AMPATH Chronic Disease Management hypertension management protocol. [file gh-15-1-856-s5.pdf]

Hypertension screening and care algorithm

Dispensary care algorithm:

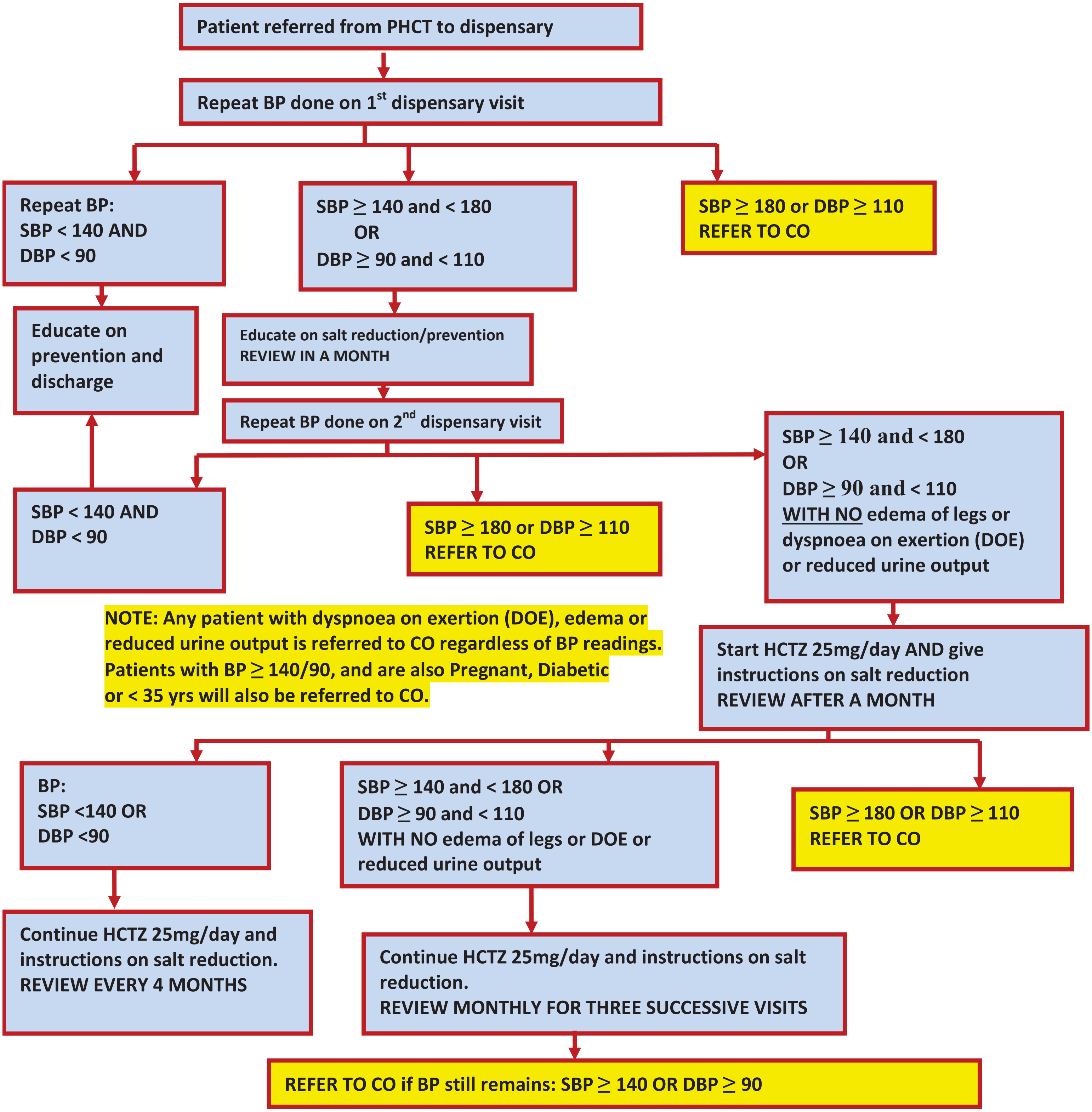

Management of hypertension in diabetes

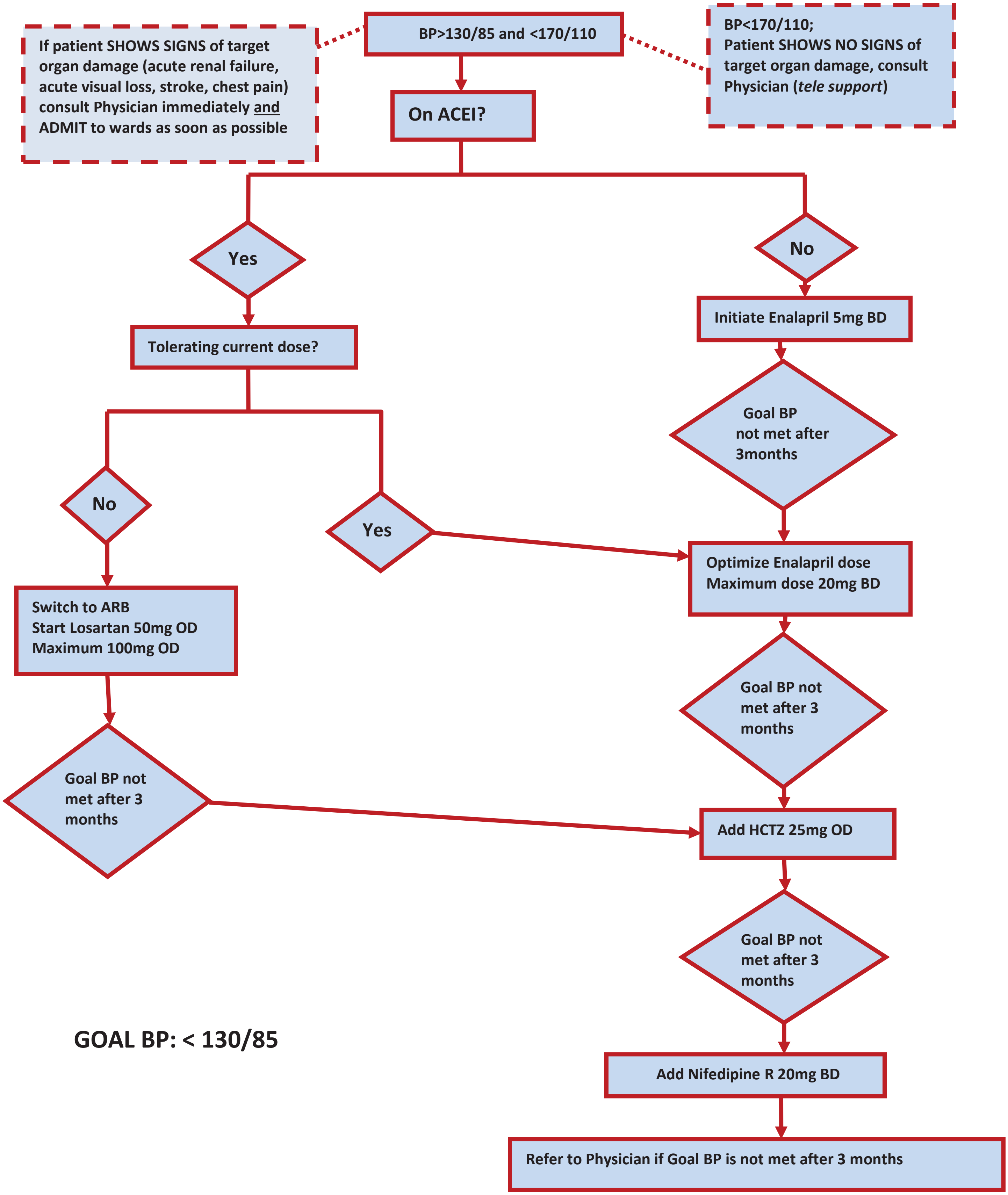

GOAL BP: < 130/85

PRICES FOR TESTS AND MEDICINES IN AMPATH CDM PROGRAM

Test	Price per test (KShs)
Creatinine	300
Hemoglobin A1C	700
Random blood sugar	150
Urinalysis	100
Blood pressure check	Free
**Medicine**	**Price per tablet (KShs)**
Amlodipine 5 mg	5
Aspirin 75 mg	2
Atenolol 50 mg	2
Atorvastatin 20mg	7
Carvedilol 6.25 mg	5
Digoxin 0.25mg	7
Enalapril 10mg	5
Enalapril 5mg	2
Furosemide 40 mg	1
Glibenclamide 5 mg	2
Hydralazine 25 mg	3
Hydrochlorothiazide 50 mg	1
Losartan 50mg	5
Metformin 500 mg	2
Metformin 850 mg	3
Nifedipine retard 20 mg	1
Propranolol 40 mg	1
Spironolactone 25mg	5
